# Supplementary material for: Spatial Variation in Stream-Water Bacterial Communities Across Sites Differing in Wild Chinese Giant Salamander Detection Frequency
Source: Biology (Basel). 2026 Sep 18;15(18):1647. doi: 10.3390/biology15181647 (PMC13604818; doi:10.3390/biology15181647)
Supplement: Supplementary file 1 [file biology-15-01647-s001.zip › Data Related to “Spatial Variation in Stream-Water Bacterial Communities across Sites Differing in Wild Chinese Giant Salamander Detection Frequency”/Microbial Data/report/src/hyperlinks/Methods_QIIME2_CH.pdf]

中文版Method

仅供客户在文章写作时参考，分析内容和方法请以结题报告
为准， 请客户自行承担文章查重等相关风险。

1 实验流程

1.1 DNA提取

根据样本类型，进行DNA提取。

1.2 PCR产物的获取

引物对应区域： 16SV4 区引物(515F和806R)： 鉴定细菌多样性。 18SV4区引物 (528F和706R)： 鉴定真核微生物多样性。ITS1区引物(ITS5- 1737F和ITS2-2043R)：鉴定真菌多样性。此外， 扩增区域还包括16SV3-V4、16SV4-V5、16SV5-V7；古菌 16SV4-V5、古菌 16SV8； 18SV9 和 ITS2 区。

所有 PCR 混合液加入 15 µL Phusion High-Fidelity PCR Master Mix、 0.2 µM 引物和10 ng基因组DNA模板， 在98℃下进行1分钟的第一次变性，然后在 98℃ (10s)、50℃ (30s) 和 72℃ (30s) 下进行30次循环， 最后在72℃下保持5分钟。

1.3 PCR产物的混样和纯化

对PCR产物进行磁珠纯化，根据PCR产物浓度进行等量混样，充分混匀后对PCR产物进行检测并回收目的条带。对扩增产生的PCR产物，进行PCR产物检测，详见样本检测报告。

1.4 文库构建和上机测序

进行文库构建，构建好的文库经过Qubit和qPCR定量，文库合格后，在NovaSeq6000或DNBSEQ-G99平台进行上机测序。

2 生物信息分析

2.1 数据质量控制

2.1.1 数据拆分

根据Barcode序列和PCR扩增引物序列从下机数据中拆分出各样本数据。

2.1.2 双端数据拼接

截去 Barcode 和引物序列后使用 FLASH（Version 1.2.11, http://ccb.jhu.edu/software/FLASH/）（Magoc T et al.,2011），对每个样本的 reads 进行拼接，得到的拼接序列为原始 Tags 数据（Raw Tags）。随后使用 Cutadapt 软件匹配反向引物序列并剪切掉余下的序列，以防止其对后续分析造成干扰。

2.1.3 数据质控

使用 fastp 软件（Version 0.23.1) 对拼接得到的 Raw Tags 经过严格的过滤处理得到高质量的 Tags 数据（Clean Tags)(Bokulich NA et al.,2012)。

2.1.4 去除嵌合体

经过以上处理后得到的Tags 需要进行去除嵌合体序列的处理，Tags 序列通过

与物种注释数据库 (Silva database, https://www.arb-silva.de/ for 16S/18S, Unite database, https://unite.ut.ee/ for ITS)进行比对检测嵌合体序列，并最终去除其中的嵌合体序列，得到最终的有效数据（Effective Tags）（Edgar RC et al.,2011）。

客户引物项目默认均与silva注释数据库（https://www.arb-silva.de/）进行比对检测嵌合体序列并去除。

若客户提供双端原始数据（raw data），将根据客户指定的扩增区域，选择对应数据库（Silva database, https://www.arb-silva.de/ for 16S/18S, Unite database, https://unite.ut.ee/ for ITS）进行嵌合体去除；若客户提供单端数据，是否去除嵌合体将根据数据类型决定：如为已质控的 clean，则不进行嵌合体去除；如为未质控的，则根据扩增区域选择对应数据库去除嵌合体。

2.2 ASV降噪和物种注释

2.2.1 ASVs降噪

对以上得到的 Effective Tags， 使用 QIIME2软件中的DADA2 模块或 deblur 进行降噪（默认使用 DADA2)，从而获得最终的 ASVs (Amplicon Sequence Variants， 即扩增子序列变异) 以及特征表(Wang Y et al.,2021)。

2.2.2 物种注释

使用 QIIME2 软件进行物种注释。对于 16S 和 18S， 数据库为 Silva 138.2，对于 ITS，数据库为 Unite v10.0。对于非常规区域，默认用 micro_NT 数据库（利用 NT库提取古菌、真菌、病毒、细菌整理得到的子库）注释。

备注：

1. 由于Silva官网序列文件SILVA_138.2_SSURef_NR99_tax_silva.fasta仅整理物种名称，缺失物种相关层级信息，同时Silva官网提供的层级信息文件不完整，因此需要通过物种名称去 ncbi获取层级。步骤是先使用Silva官网的层级信息补充物种层级，对未定位到物种层级的物种信息则使用 ncbi 官网提供的 dmp 文件进行层级补充。

2. Micro_NT 库注释原理：将序列与Micro_NT 数据库中的序列进行blast比对，获取该序列与数据库中各序列打分前 20 的结果，按照 bit score 最大值进行筛选，随后使用 LCA 算法来推断该序列所属的最近共同祖先。NT数据库中大量的未分类信息，导致注释效果大大降低，注释的结果会出现较多的未分类 Unclassfied。为保证数据的精确性，我们在进行最近公共祖先算法获取物种信息时，会忽略 Unclassfied 未分类物种，降低其对注释造成的影响。

2.2.3 构建系统发育树

使用QIIME2软件进行快速多序列比对，得到所有ASV序列的系统发生关系。

2.2.4 数据均一化

最后对各样本的数据进行均一化处理，以样本中数据量最少的为标准进行均一化处理，后续的Alpha多样性分析和Beta多样性分析都是基于均一化处理后的数据。

2.2.5 物种丰度统计

根据每个样本在不同分类等级（门、纲、目、科、属、种） 的丰度前10的物种，

通过SVG 函数绘制Perl中相对丰度的分布直方图。

2.2.6 热图

利用每个分类级别的丰度前35的物种丰度信息绘制热图，直观地显示了不同的丰

度和分类群聚类。这是用R的pheatmap()函数实现的。

2.2.7 三元相图

根据每个分类级别的前10个分类群的三元图可以用来显示三个样本之间的丰度差异。它是用R的vcd()函数中计算的。

2.2.8 韦恩图和花瓣图

Venn和Flower图直观地显示了不同样本或组之间的共同和独特信息。Venn图和Flower图分别用 VennDiagram() 函数在R中生成，用 SVG 函数在perl中生成。

2.2.9 系统进化分析

系统发育树，也称为进化树，可以描述不同物种之间的进化关系。选择样本中丰度最高的100个属，进行序列比对，用perl绘制SVG格式的系统发育树。

2.3 样本复杂度分析（Alpha Diversity）

2.3.1 Alpha多样性指数分析

使用QIIME2 软件计算observed_otus 、 shannon 、 simpson 、 chao1 、 goods_coverage 、dominance 和pielou_e指数。Alpha多样性指数具体描述如下：

Observed_otus – (http://scikit-bio.org/docs/latest/generated/skbio.diversity.alpha.observed_otus.html);

Chao1 – (http://scikit-bio.org/docs/latest/generated/skbio.diversity.alpha.chao1.html#skbio.diversity.alpha.chao1);

Dominance – (http://scikit-bio.org/docs/latest/generated/skbio.diversity.alpha.dominance.html#skbio.diversity. alpha.d ominance)

计算菌群多样性(Community diversity) 的指数有：

Shannon – (http://scikit-bio.org/docs/latest/generated/skbio.diversity.alpha.shannon.html#skbio.diversity.alpha.shannon);

Simpson – (http://scikit-bio.org/docs/latest/generated/skbio.diversity.alpha.simpson.html#skbio.diversity.alpha.simpson);

计算测序深度的指数有：

Coverage – (http://scikit-bio.org/docs/latest/generated/skbio.diversity.alpha.goods_coverage.html#skbio.diversity.alpha.goods_coverage);

计算物种均匀度的指数有：

Pielou_e – (http://scikit-bio.org/docs/latest/generated/skbio.diversity.alpha.pielou_e.html#skbio.diversity.alpha.pielou_e).

2.3.2 物种累积箱型图

为了评估微生物群落的丰富度和样本量。物种积累箱图可以用于可视化，这是用R包执行的。

2.3.3 等级梯度曲线

通过观察曲线的宽度和形状来反映样本物种的丰富度和均匀度。这可以使用R中的ColorBrewer 软件包绘制。

2.3.4 稀释曲线

测序数量不足可能导致样本信息不足，而过多的测序深度也可能导致不必要的成本增加。因此，确定合适的测序量是至关重要的。绘制稀疏曲线提供了发现序列深度是否足够的可行性的能力。 这是通过使用plyr包的R来实现的。

2.4 多样本比较分析（Beta Diversity）

2.4.1 Beta多样性分析

为了评估群落组成的复杂性并比较样本(组)之间的差异，在QIME2中，基于的加权和非加权距离进行β多样性分析

2.4.2 Beta多样性热图

β多样性分析用于评估样本在物种复杂性方面的差异。通过QIIME2软件计算加权和未加权unifrac的β多样性距离。然后绘制一个热图来显示样本之间的unifrac距离，这是用Perl实现的。

2.4.3 UPGMA聚类分析

基于加权unifrac距离矩阵，在UPGMA上构造了聚类树。这在生态学中被广泛用于进化分类。UPGMA图是通过QIIME2中的UPGMA.tre函数绘制的。

2.4.4 降维分析

主成分分析（PCA），该分析用于使用带有R软件的ade4软件包和ggplot2软件包（4.0.3版）来降低原始变量的维数。

主坐标分析（PCoA）用于从复杂和多维数据中获取主坐标并进行可视化。在转换到一组新的正交轴之前，获得了样本之间加权或未加权均匀性的距离矩阵，通过该矩阵，第一主坐标表示最大变化因子， 第二主坐标表示第二大变化因子。PCoA分析通过R软件（4.0.3版） 中 的ade4包和ggplot2包计算和绘制。

非度量多维度分析（NMDS）来降低数据维度。与PCoA类似，NMDS也使用距离矩阵，但它强调的是数值秩。图上样本点之间的距离只能反映秩信息， 而不能反映数值差异。NMDS 分析是通过带有ade4软件包和ggplot2软件包的R软件实现的。

2.5 群落差异分析

通过Anosim、Adonis、MRPP、Simper、T检验、Metagenomeseq和LEfSe等一系列统计分析，揭示了群落结构的分化。

Anosim、Adonis和MRPP分析是分析高维数据组之间差异的非参数检验。这可

以分析分组之间的差异是否显著大于组内的差异，这可以确定分组是否有意义。这些可以在R软件内使用vegan包和ggplot2包进行分析和绘制。

Simper可以揭示每个物种对群体之间分化的贡献。选出了前10个物种，并将其显示在图表上。这在R中使用Vegan软件包和ggplot2软件包进行。

Metagenomeseq可以展示群体之间表现出显著差异的物种。在R中使用MetagenomeSeq包进行。

LEfSe被广泛用于发现生物标志物，它可以揭示宏基因组特征。这是lefse的单独软件进行计算和绘制的。

2.6 功能预测

PICRUSt（V1.1.4）主要用于预测基于标记基因的宏基因组功能。PICRUSt2（V2.3.0）是 PICRUSt的改进版本。

Tax4Fun（V0.3.1）是一个R软件，广泛用于肠道和土壤样本。总的来说，与PICRUSt相比，它可以提供更准确的结果，尤其是对于土壤样本。

BugBase工具，可以发现显微组织的表型。它可以根据七种表型对微生物群落进行分类：革兰氏阳性、革兰氏阴性、生物膜形成、致病性、含移动元素、氧气利用（包括好氧、厌氧和可培养厌氧）和氧化应激耐受性。

在处理真菌样本时，可以使用FunGuild工具，对主要的营养类型进行分类，研究具体的真菌功能分类。

FAPROTAX是主要阐明生物化学过程和元素时发挥重要作用的软件。

2.7 关联分析

2.7.1 网络图

为了探索物种之间的共生关系，揭示环境因素对群落结构的影响，绘制了二维和三维网络图进行可视化。

2.7.2 环境因子分析

可以使用spearman相关性检，CCA/RDA和dbRDA等进一步分析来反映环境因素与物种丰度之间的相关性。所有这些图表和分析都是在R中完成的。

3 参考文献

Bokulich NA, Subramanian S, Faith JJ, et al. Quality-filtering vastly improves diversity estimates from Illumina amplicon sequencing. Nature Methods. 2012;10(1):57-59. doi:10. 1038/nmeth.2276.

Edgar RC, Haas BJ, Clemente JC, Quince C, Knight R. UCHIME improves sensitivity and speed of chimera detection. Bioinformatics. 2011;27(16):2194-2200. doi:10. 1093/bioinformatics/btr381.

Magoc T, Salzberg SL. FLASH: fast length adjustment of short reads to improve genome assemblies. Bioinformatic. 2011; 27(21): 2957-2963. doi: 10. 1093/bioinformatics/btr507.

Wang Y, Guo H, Gao X, Wang J. The Intratumor Microbiota Signatures Associate with Subtype, Tumor Stage, and Survival Status of Esophageal Carcinoma. Frontiers in Oncology. 2021;11. doi:10.3389/fonc.2021.754788
